# Towards Botanical Authentication of Ginkgo Food Supplements: A Holistic Approach Based on Phytochemical and Genomic Markers

**DOI:** 10.3390/foods14173111

**Published:** 2025-09-05

**Authors:** Liliana Grazina, Paula Paíga, Joana S. Amaral, Joana Costa, Manuela M. Moreira, Cristina Delerue-Matos, Isabel Mafra

**Affiliations:** 1 REQUIMTE/LAQV, Faculdade de Farmácia, Universidade do Porto, Rua de Jorge Viterbo Ferreira 228, 4050-313 Porto, Portugal; lggrazina@gmail.com (L.G.); jbcosta@ff.up.pt (J.C.); 2 REQUIMTE/LAQV, Instituto Superior de Engenharia do Porto, Instituto Politécnico do Porto, Rua Dr. António Bernardino de Almeida 431, 4249-015 Porto, Portugal; paula.paiga@gmail.com (P.P.); mmdsm@isep.ipp.pt (M.M.M.); cmm@isep.ipp.pt (C.D.-M.); 3 CIMO, LA SusTEC, Instituto Politécnico de Bragança, Campus de Santa Apolónia, 5300-253 Bragança, Portugal

**Keywords:** *Ginkgo biloba*, plant food supplements, leaf extracts, real-time PCR, LC-MS, authenticity

## Abstract

*Ginkgo biloba* is one of the most consumed medicinal plants and broadly included as an ingredient in plant food supplements (PFS) and herbal infusions, being potential targets for economically motivated adulteration. This work aimed at comparing the use of DNA and phytochemical markers to authenticate the botanical origin of ginkgo-leaf extracts and PFS. Quantitative real-time PCR was used to detect ginkgo DNA, while ultra-high performance liquid chromatography with tandem mass spectrometry detection (UHPLC-MS/MS) determined its main phytochemicals (terpene lactones and flavonol aglycones). DNA was detected in all ginkgo leaf extracts, mainly water, while the highest levels of phytochemicals were obtained using ethanol or acetone as solvents. The results suggested that 4 out of a total of 19 PFS samples were adulterated, with two samples evidencing the addition of quercetin from sources other than ginkgo. The other two samples showed low amounts of ginkgo phytochemicals, which was corroborated by low DNA content, suggesting the use of reduced amounts of *G. biloba* leaf material.

## 1. Introduction

*Ginkgo biloba* is one of the most popular botanicals used for medicinal purposes, being widely included as an ingredient in several herbal products, such as plant infusions, and dietary supplements or plant food supplements (PFS) [1,2]. Its main pharmacologically active compounds are flavonol glycosides and terpene tri-lactones (bilobalide and ginkgolides) [3,4]. While flavonol glycosides are the most abundant, terpene lactones are unique to ginkgo and are believed to have the ability to cross the blood–brain barrier due to their lipophilic character, thus being capable of acting on the central nervous system [5]. Additionally, ginkgo extracts possess antioxidant, antineuro-inflammatory, anti-asthmatic, and wound healing properties [3,5]. Therefore, these phytochemicals are thought to improve brain function and alleviate cognitive impairment in the elderly, providing a therapeutic action in minor peripheral circulatory disorders and being indicated to vascular dementia [1,3,6].

The large demand for *G. biloba* leaf in the global market and the widespread consumption of ginkgo herbal products makes this plant a potential target for economically motivated adulteration by substitution with other lower value plant species, such as *Styphnolobium japonicum*, which is also rich in flavonol glycosides. Moreover, the addition of synthetic phytochemicals, mainly flavonol aglycones, is also a practice reported in counterfeit ginkgo-containing PFS [7,8]. While adulteration practices involving the mixture or substitution of plant material with other species can be detected by DNA-based techniques, the addition of synthetic or foreign chemical compounds can only be detected by chemical approaches. Thus, different adulteration practices may require distinct or complementary analytical tools since the accuracy and reliability of assessing the authenticity of herbal products greatly depend on the limitations of the applied methodology. DNA-based methods have been previously applied to assess the authenticity of ginkgo-containing products. Dhivya et al. [9] proposed a probe-specific real-time PCR method to identify ginkgo DNA in plant-based products, but its applicability to analyze commercial products was not demonstrated. Little [10] developed a DNA barcoding assay targeting the matk gene to successfully authenticate PFS. Liu et al. [11] proposed a rapid method based on recombinant polymerase amplification in a lateral flow device to detect ginkgo and its adulterant in ginkgo-containing products, showing promising results, but with limited cross-reactivity tests to confirm specificity. More recently, Grazina et al. [12] developed and validated a real-time PCR method to detect and quantify *G. biloba* in herbal mixtures, which was successfully applied to several commercial ginkgo-containing herbal infusions.

Chemical approaches relying on the analysis of ginkgo bioactive compounds have also been reported, targeting terpene lactones and flavonoids, whose contents in the standardized extract of *G. biloba* are established by the European Pharmacopeia [13]. Due to the lack of UV absorption by terpene lactones, many studies have focused on the flavonoid group, and various pharmacopeias have recommended a straightforward assay based on high performance liquid chromatography (HPLC) with UV detection to quantify the three major aglycones, namely quercetin, kaempferol and isorhamnetin, after acid hydrolysis [14]. However, this simple approach has shown limitations, particularly when adulteration is carried out by the addition of pure aglycones. Other studies have proposed the use of liquid chromatography coupled to mass spectrometry (LC-MS) to determine various characteristic terpene lactones, as marker compounds of ginkgo [15,16]. However, in cases of partial substitution of ginkgo with other species, additional analytical approaches are required to ensure accurate identification. To address this, targeted profiling of both terpene lactones and flavonoids has been implemented, offering a more comprehensive and reliable analysis [14]. To this end, LC-MS with a single quadrupole operating in single ion monitoring (SIM) was used to quantify ginkgolides A, B, C, bilobalide, quercetin, kaempferol, isorhamnetin and three quercetin glycosides [17]. Similarly, Demirezer et al. [18] used this technique to quantify ginkgolides A, B, C and J, and the same aglycones. Liu et al. [19] targeted terpene lactones as markers in combination with the three major aglycones and ten flavonol glycosides, achieving higher selectivity and sensitivity through a triple quadrupole (QQQ-MS/MS) operating in multiple reaction monitoring (MRM) mode. Wang et al. [20] employed ultra performance liquid chromatography (UPLC) with QQQ-MS/MS in MRM mode, though their analysis was restricted to four terpene lactones, four biflavonoids and three flavonols, limiting its ability to detect possible adulteration with pure aglycones. In addition, high-resolution mass spectrometry (HR-MS) has been applied for the authentication of *G. biloba*, either through the qualitative identification of multiple compounds based on accurate mass data [21,22] or via an untargeted approach to establish chemical fingerprints, which were subsequently analyzed using chemometrics for pattern comparison [23].

In this study, a comparative assessment of two approaches for authenticating the botanical origin of ginkgo-containing products (in-house dried ginkgo leaf extracts and PFS) was conducted, namely real-time PCR and liquid chromatography coupled to mass spectrometry. In the first approach, the fate of ginkgo DNA along the preparation of dry-leaf extracts obtained with different extraction protocols was investigated using the method previously developed by Grazina et al. [12]. For the second approach, an ultra-high performance liquid chromatography with tandem mass spectrometry (UHPLC-MS/MS) method was optimized and validated for the simultaneous analysis of flavonols and terpene lactones [21,24]. Both approaches were further applied to analyze commercial PFS, and the obtained results were compared and critically assessed for their complementarity. To the best of our knowledge, the use of an orthogonal approach based on DNA analysis and phytochemical profiling is herein proposed for the first time to assess the authenticity of ginkgo PFS.

## 2. Materials and Methods

### 2.1. Reagents

For UHPLC-MS analysis, methanol was supplied by J.T. Baker (Deventer, Netherlands) and acetonitrile and propanol were obtained from Sigma-Aldrich (Steinheim, Germany). Ultra-pure water (18.2 MΩ/cm) was produced using a Simplicity 185 system (Millipore, Molsheim, France). All chromatographic solvents were filtered through a 0.22 μm nylon membrane filter (Supelco, Bellefonte, PA, USA) using a vacuum pump (Dinko D-95, Barcelona, Spain) and degassed for 15 min in an ultrasonic bath (Sonorex Digital 10P, BANDELIN, Germany). Formic acid (≥95%) was purchased from Sigma-Aldrich (St. Louis, MO, USA) and acetic acid was acquired from Carlo Erba (Val-de-Reuil, França). Ginkgolide A, ginkgolide B, ginkgolide C, ginkgolide J, (-)-bilobalide, isorhamnetin, kaempferol, quercetin dihydrate, and the internal standard andrographolide were of high purity grade (≥95%) and were acquired from Extrasynthese (Genay, Lion, France).

### 2.2. Ginkgo-Leaf Extracts and Commercial Samples

*G. biloba* leaves were kindly provided by the Botanical Garden of University of Porto (Porto, Portugal), Serralves Garden (Porto, Portugal), Botanical Garden of Bern (Bern, Switzerland), and Botanical Garden of Madeira (Madeira, Portugal). Different protocols for the extraction of phytochemicals from *G. biloba* leaves were tested in-house using several solvent formulations: water, ethanol in water (70%, 50%, and 30%, *v/v*), and acetone in water (60%, *v/v*). The extractions were carried out using 10 g of dried and ground ginkgo leaves in 100 mL of solvent at 37 °C with stirring using an orbital incubator at 160 rpm, overnight. The solutions were filtered, evaporated in a rotatory evaporator and lyophilized, obtaining ginkgo leaf dried extracts.

Additionally, 19 commercial samples, including 8 solid and 11 liquid PFS, were acquired in local specialized stores (Porto, Portugal) and in the web market, and their relevant label information is described in Table 1.

### 2.3. DNA Analysis

#### 2.3.1. DNA Extraction

DNA extraction was performed from 100 mg of lyophilized ginkgo leaf extracts or ground solid PFS samples (Table 1) using the NucleoSpin Plant II kit (Macherey-Nagel, Düren, Germany), following protocol B, with slight modifications as described by Costa et al. [25]. Liquid samples of PFS (Table 1) were prepared by adding 5 mL of absolute ethanol to 5 mL of sample, except for sample #14 that was prepared using 10 mL of absolute ethanol and 10 mL of sample. The mixtures were stirred for 15 min at 160 rpm, centrifuged for 15 min at 7000× *g* and the remaining pellet submitted to DNA extraction as described above. All the extracts were kept at –20 °C until further analysis.

Yield and purity of DNA extracts were assessed by UV spectrophotometry using a Take3 micro-volume plate accessory on a Synergy HT multi-mode microplate reader (BioTek Instruments, Inc., Winooski, VT, USA). The nucleic acid protocol with sample type defined for double-strand DNA in the Gen5 data analysis software version 2.01 (BioTek Instruments, Inc., Winooski, VT, USA) was used for measuring absorbance data at 260 and 280 nm.

#### 2.3.2. Qualitative PCR and Real-Time PCR

Firstly, the DNA extracts were analyzed by PCR with universal primers targeting a eukaryotic region (EG-F/EG-R) [26] to confirm their amplification capacity. A species-specific PCR assay targeting the ITS1 region of *G. biloba* was performed using the primers Gkb2-F/Gkb2-R and the conditions described by Grazina et al. [12]. PCR amplifications were carried out in a MJ Mini™ Gradient Thermal Cycler (Bio-Rad Laboratories, Hercules, CA, USA). PCR products were analyzed by electrophoresis in a 1.5% agarose gel containing 1× Gel Red (Biotium, Fremont, CA, USA) for staining and carried out in 1× SGTB buffer (GRISP, Porto, Portugal) for 20–25 min at 200 V.

Real-time PCR assays were performed using the ginkgo-specific primers (Gkb2-F/Gkb2-R) and probe (Gkb2-P), following the conditions and temperature program described by Grazina et al. (2020) [12] in a fluorometric thermal cycler CFX96 Real-time PCR Detection System (Bio-Rad Laboratories, Hercules, CA, USA). Data analysis was performed using the Bio-Rad CFX Manager 3.1 (Bio-Rad Laboratories, Hercules, CA, USA) software. The calibration curve was constructed using a 10-fold serially diluted ginkgo DNA extract (50 ng–0.005 pg).

### 2.4. Phytochemical Analysis

#### 2.4.1. UHPLC-MS/MS Analysis

UHPLC-MS/MS analysis was carried out in a Shimadzu Nexera UHPLC system (Shimadzu Corporation, Kyoto, Japan) equipped with two pumps (LC–30 AD) for the solvent delivery, a column oven (CTO–20 AC) and an auto-sampler (SIL–30 AC) coupled to a Shimadzu LCMS–8030 triple-quadrupole mass spectrometer (Shimadzu Corporation, Kyoto, Japan) operating in the electrospray ionization (ESI) mode. The system was controlled by a CBM–20A module. Lab Solutions software (Shimadzu Corporation, Kyoto, Japan) was used for the control and data processing. The chromatographic analysis was performed using a Cortecs^®^ UPLC^®^ C18+ (1.6 µm particle size, 2.1 mm ×100 mm) (Waters, Milford, MA, USA). A flow rate of 0.3 mL/min and 5 µL injection volume were used. The column oven was set at 30 °C and the auto-sampler operated at 4 °C. Argon was used as the collision-induced dissociation gas at a pressure of 230 kPa.

The precursor ion and ionization mode for each compound were selected by recording chromatograms for each standard in a full scan mode. Scan spectra were performed in Q3 and Q1 quadrupoles in positive and negative ionization modes. The MS was operated in MRM mode and two transitions were monitored for each compound, except for ginkgolide J for which only one transition was monitored. The most intense transition was used as quantifier and the second transition was used as qualifier.

The ion source parameters were optimized by direct injection of a standard mixture solution (10 mg/L). For both ionization modes, the obtained results were set as follows: heat block and desolvation line temperatures of 500 °C and 300 °C, interface voltage of 5 kV, and drying gas and nebulizing gas flows of 10 and 2.0 L/min, respectively.

The optimal chromatographic conditions for positive ionization mode (ESI+) for the analysis of ginkgolide J and ginkgolide A were set using a mobile phase of 0.1% aqueous formic acid as eluent A and methanol as eluent B, operating with a linear gradient starting from 10 to 100% of eluent B in 3 min, with a hold of 3 min. Then, the initial conditions were reached in 1 min and maintained for 4 min before the next run, corresponding to a total analysis time of 11 min. Regarding the other compounds under study (isorhamnetin, kaempferol, quercetin dihydrate, bilobalide, ginkgolide C and ginkgolide B), the chromatographic conditions for the negative ionization mode (ESI–) were set using a mobile phase of 0.05% aqueous acetic acid as eluent A and methanol as eluent B. A linear gradient starting with 5% and increased to 100% of eluent B in 3 min was used, which was maintained during 2 min, decreasing to the initial conditions (5% B) in 1 min, and held during 4 min before the next injection (total analysis time of 10 min). Once the chromatographic programs in ESI+ and ESI– modes were optimized, the dwell time was evaluated. Dwell times of 100 and 60 ms were selected for ESI+ and ESI– modes, respectively. The internal standard andrographolide was analyzed in both ionization modes.

#### 2.4.2. Sample Preparation and Extraction of Phytochemicals

For the UHPLC-MS/MS method development, a representative mixture of all solid PFS (Table 1) was obtained by grinding and mixing the same amount of each sample. Several tests to improve the extraction process were carried out according to the procedures described by Ding et al. [17] and Könczöl et al. [27]. Accordingly, the following variables were tested: volume of extraction solvent for 100 mg of sample (5, 10, and 20 mL), extraction time (5, 10, 15, 25, and 50 min), occurrence of precipitation, loss of target compounds during the evaporation step, and pre-concentration factor.

For the solid samples, 100 mg of ground material were mixed with 10 mL of methanol with vortexing. The mixture was placed in an ice bath and extracted by sonication using an ultrasonic processor (VCX750, Sonics & Materials, Inc., Newtown, CT USA), at room temperature for 5 min. Then, it was centrifuged for 10 min at 4500 rpm, and the supernatant was filtered using a syringe filter PTFE 0.22 µm (13 mm, BGB, Switzerland). For liquid samples, a dilution step was firstly performed as described by Könczöl et al. [27].

Each extract of solid and liquid samples was injected directly and after 2-, 5-, 10-, 25- and 50-fold dilution, considering sample variability of the compounds under analysis. Afterwards, 10 µL of the internal standard (10 mg/L) were added to the standard solutions and to the samples (extracts and respective dilutions) before the injection into the UHPLC-MS/MS. For each sample, three extractions were performed, and each extract was injected three times.

#### 2.4.3. Method Validation

Botanical species contain several compounds, such as polysaccharides, phenolic compounds [28], among others, that can inhibit or enhance the signal intensity of the analytes under study [29,30]. The matrix effect (ME) was determined as the ratio of the area of a standard prepared in the matrix and the area of the standard with the same concentration prepared in solvent (methanol). A blank sample (without addition of standards) was simultaneously assayed to subtract the area of the target analyte present in the sample to the area of the target analyte present in the fortified sample. If there is no ME, the ratio calculated was 100%. On the other hand, if this ratio is above or below 100%, it reflects ion enhancement or suppression, respectively. ME was determined in the extracts obtained from solid, liquid and lyophilized samples, as well as in the respective dilutions. Recoveries were calculated by comparing the area of the target compound in the sample fortified before the extraction (pre-spiked sample) with the area of the target compound in the sample fortified after the extraction (post-spiked sample). Calibration curves were obtained using linear regression analysis and eleven levels ranging from 1 to 1000 µg/L of standard compounds. Quantification of the compounds was performed by the internal standard approach. The limits of detection (LOD) and quantification (LOQ) were calculated as 3 and 10 times the signal-to-noise ratio. LOD and LOQ were determined for each compound across the different types of samples analyzed, as variations in sample matrices, analyte properties and extraction methods, which can significantly affect the sensitivity. According to the described in the guidance document on the estimation of LOD and LOQ for measurements in the field of contaminants in feed and food, the estimate of LOD and LOQ may vary among sample matrices and, therefore, must be established for each specific matrix [31]. The precision of the method, expressed as the relative standard deviation (RSD%), was obtained by successive injection of two concentrations of the standard mixture (100 and 1000 µg/L) at the beginning and at the end of each batch sequence.

### 2.5. Statistical Analysis

The statistical analysis was performed using the GraphPad Prism version 8.0.2 (GraphPad Software, San Diego, CA, USA). Data were firstly evaluated for normal distribution using the Shapiro–Wilk test. Data with normal distribution were analyzed by ordinary one-way ANOVA following Tukey multiple comparison test, while the non-normal distribution was submitted to non-parametric Kruskal–Wallis test following Dunn’s multiple comparison test to assess the significance of differences among the terpenes lactones and flavonol aglycones contents as affected by solvent extraction method. Significant statistical differences were considered when *p* < 0.05.

## 3. Results and Discussion

### 3.1. DNA Analysis

#### 3.1.1. Quality of Extracted DNA

Generally, isolated DNA obtained from dried ginkgo leaf extracts showed sufficient yields, being mostly within 36.7–321.5 ng/µL. The exceptions were for the ginkgo leaf extracts obtained with higher proportions of organic solvents. The purities of DNA extracts, expressed as the ratio of the absorbances at 260 nm and 280 nm (A260/A280), ranged from 1.5 to 2.2, suggesting generally acceptable/high-quality DNA. The amplification capacity was further confirmed by PCR amplification with primers targeting a eukaryotic 18S rRNA nuclear gene [26] since all extracts produced the expected 109 bp amplicon. However, ethanolic and aqueous lyophilized extracts showed poor stability and reproducibility in DNA extraction.

Concerning the samples of PFS, the DNA yields were generally low, being within 10.5–167.0 ng/µL for solid supplements and 6.0–7.0 ng/µL or not quantifiable (below UV/Vis spectrophotometric detection) for liquid formulations. Regarding purity, the DNA extracts from solid PFS were mostly within the acceptable range (1.7–2.0), except for samples #4 and #8 with purities lower than desired (1.3–1.4). About liquid PFS, as expected, the values of purities (0.85–1.78) were, in general, low and mostly below 1.7. The high variability in the yield and purity of DNA extracts of both solid and liquid formulations was probably due to the presence of excipients, which could interfere with the DNA extraction process [25].

#### 3.1.2. Analysis of Ginkgo Leaf Extracts and PFS

**Figure 1:**
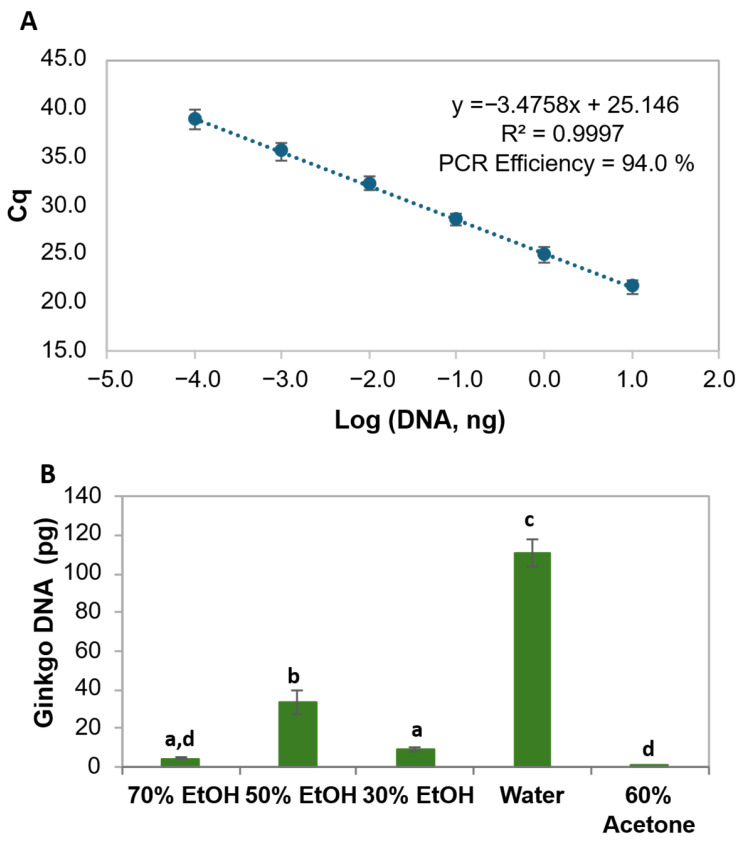
Calibration curve of real-time PCR with a hydrolysis probe targeting the ITS1 region of *G. biloba* using 10-fold serially diluted ginkgo DNA (20 ng to 0.2 pg) (n = 10 replicates) (**A**). Mean ginkgo DNA content $\left(DNA(ng)=10^{\left(\frac{C_{q}-b}{m}\right)}\right)$ ± standard deviation (SD) (n = 4 replicates from 2 independent assays) of ginkgo leaf extracts obtained with different solvents: three ethanol/water (EtOH) concentrations (30, 50 and 70%), 60% of acetone/water (**B**). Different letters mean statistically significant differences among the extraction methods (*p* < 0.05), following ordinary one-way ANOVA. Cq, cycle of quantification.

Currently, numerous processes are available for preparing *G. biloba* leaf extracts. While these processes are well established for medicinal purposes, ensuring regulated levels of flavonols and terpenes in the final product, the same does not apply to food supplements. Although herbal medicines should comply with pharmacopeia monographs, there is significant variation particularly in what concerns the primary extraction of the crude material [32]. While the European Pharmacopeia indicates the mandatory use of acetone:water (6:4 *v/v*) and the Chinese Pharmacopeia allows only hydroethanolic extraction, the US Pharmacopeia is more flexible on the extraction solvents, allowing the use of acetone-water or other suitable solvents as long as the final specifications are fulfilled [31]. Moreover, one of the most known and used extracts worldwide is the proprietary extract EGb 761^®^, obtained by a complex process involving a primary extraction with acetone 60% [31]. Therefore, to accommodate the wide variability accepted in the production of *G. biloba* extracts, which can be even broader in the food supplement industry, in this work, several protocols were tested to cover the most commonly used primary extraction solvents, namely hydroethanolic mixtures and acetone 60%. Additionally, as water is an affordable and non-toxic solvent increasingly considered for the extraction of bioactive compounds, it was also included in the study. All the dried ginkgo leaf extracts were successfully amplified by qualitative PCR and real-time PCR targeting the ITS1 region of *G. biloba*, with quantification cycles (Cq) ranging from 27.98 to 38.70. To estimate the DNA content in each leaf extract, a calibration curve was constructed using 10-fold serially diluted ginkgo DNA (20 ng to 0.2 pg) (Figure S1, Supplementary Materials) and the analytical performance criteria of Bustin et al. [33] and ENGL [34] were carefully considered. The calibration curve constructed with the mean data of all the assays (Figure 1A) exhibited parameters meeting the acceptable criteria for real-time PCR assays (PCR efficiency within 90–110%, the slope from −3.6 to −3.1 and the R2 > 0.98). The LOD was established as the lowest amount that can be reliably detected with a level of confidence of 95%, ensuring ≤5% of false negative results, while the LOQ was the lowest amount included in the dynamic range [34]. Accordingly, 0.2 ng (Cq = 38.92) of ginkgo DNA was established both as the LOD and LOQ. The linear dynamic range of the calibration curve covered 5 orders of magnitude (Figure 1A), which was according to the requirements of at least 3 orders of magnitude that ideally should extend to 5 or 6 log10 concentrations [33]. Therefore, the obtained calibration curve was applied to estimate the DNA content in each extract, whose results are displayed in Figure 1B. The water ginkgo leaf extracts provided the highest levels of DNA amplification with values (98.5–122.0 pg) significantly different (*p* < 0.05) from all the others, followed by the 50% ethanol extracts (25.2–58.7 pg), which also differed statistically. The 30% ethanol extracts (8.68–10.15 pg) do not differ statistically from the 70% ethanol extracts (3.81–6.45 pg), while acetone extracts, having the lowest DNA yields (0.36–0.41 pg), did not differ significantly from the 70% ethanol extracts. These results agree with the higher affinity of DNA molecules to more hydrophilic solvents rather than organic solvents such as acetone. The fact that several companies manufacture *G. biloba* leaf extracts using acetone:water (*v/v*) mixtures may explain the difficulties faced in the extraction and amplification of ginkgo DNA from some PFS samples (Table 1). Nevertheless, despite the complexity of the formulations, it was possible to extract amplifiable DNA from all the PFS samples, except for three (#3, #10 and #16), which might be attributed to the low amount of ginkgo material, the presence of excipients or/and the producing process (Table 1). Generally, the solid samples (#1–#8) provided higher DNA yields than the liquid formulations, with Cq values within 22.84–32.06 that corresponded to 6.1 ng–10.4 pg- of ginkgo DNA, comparing with the liquid formulations (#9-#19) that exhibited poor amplification rates (Cq within 33.14–38.82, corresponding to 0.22–4.65 pg of ginkgo DNA). Therefore, from the analyzed PFS, 16 proved to be according to the labeled ginkgo species, while three samples provided an inconclusive result that should be further analyzed by chemical approaches.

### 3.2. Phytochemical Analysis

#### 3.2.1. Optimization of UHPLC-MS/MS Conditions

Initially, the precursor and product ions and the ionization mode were determined (see Section 2.4) using solutions of the individual compounds to establish the best conditions for specificity and sensitivity. For that purpose, the MRM transitions of analytes were monitored in the positive and negative ESI modes. Almost all analytes under study presented intense ions in the negative ionization mode, except for ginkgolide J and ginkgolide A, which were analyzed in the positive ionization mode (Table S1). All the compounds presented two MRM transitions, except for ginkgolide J that exhibited a poor fragmentation and only one transition (Table S1). The internal standard, andrographolide (10 mg/L) was monitored in both ionization modes.

**Figure 2:**
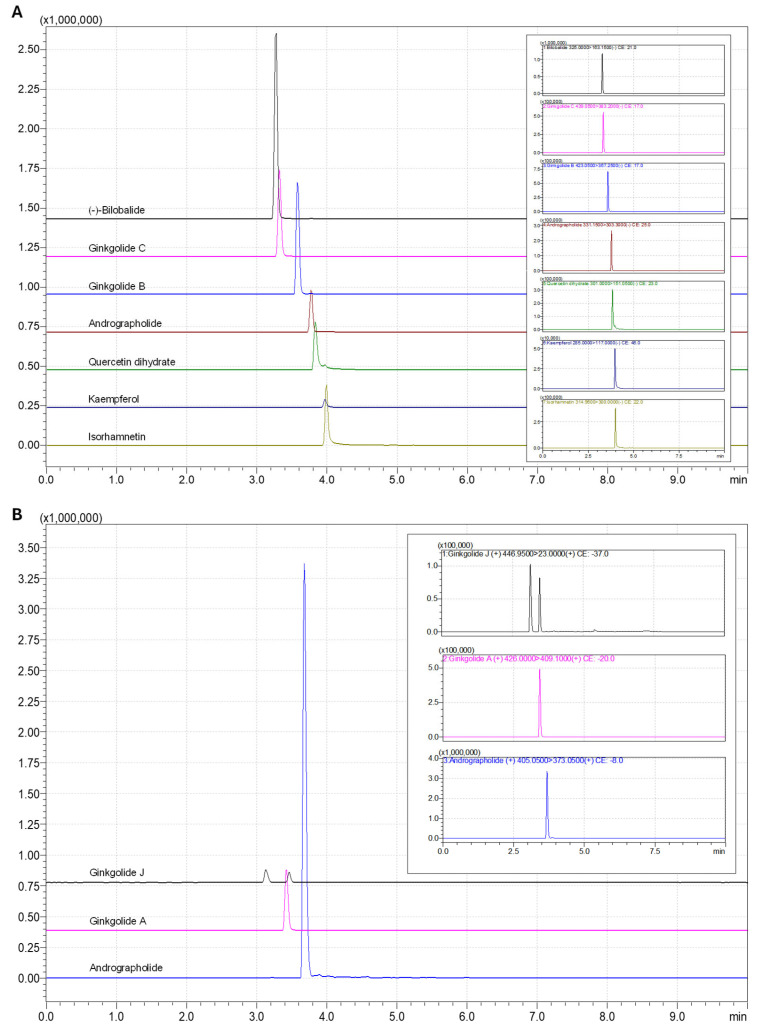
Overlaid and individual chromatograms of the compounds analyzed in the negative ionization ((-)-bilobalide, ginkgolide C, ginkgolide B, andrographolide, quercetin dihydrate, and kaempferol) (**A**) and positive ionization (andrographolide, ginkgolide J, and ginkgolide A) (**B**) modes. The concentration of the analyzed compounds was 1 mg/L, except for andrographolide (10 mg/L).

After selecting the most appropriate ions, the ion source parameters were optimized for both ionization modes regarding the drying and nebulizing gas flow rates, heat block and desolation temperatures, and interface voltage (Figures S2 and S3) to select the best operating conditions. A total of 35 tests were performed in each ESI mode, with three injections per test. The experiments were distributed as follows: 7 tests to evaluate heat block temperature (200–500 °C, 50 °C increments), 5 tests for desolvation line temperature (200–300 °C, 25 °C increments), 10 tests for interface voltage (0.5–3.0 kV, 0.5 kV increments), 4 tests for drying gas flow (10–17 L/min, 2.5 L/min increments from 10 to 15 L/min), and 9 tests for nebulizing gas flow (0.5–3.0 L/min, 0.5 L/min increments from 0.5 to 2.0 L/min and 0.2 L/min increments from 2.0 to 3.0 L/min). Figures S2 and S3 shows the effects of ion source parameters on the signal of the target compounds. These included andrographolide, ginkgolide A, and ginkgolide J in positive ionization mode, and andrographolide, (−)-bilobalide, quercetin dihydrate, kaempferol, ginkgolide B, ginkgolide C, and isorhamnetin in negative ionization mode. For the compounds analyzed in positive ionization mode, increases in heat block and desolvation line temperatures gradually increased signal intensity, with optimal responses observed at higher values. Interface voltage had a pronounced effect, as signal areas increased steadily with higher voltages, indicating enhanced ionization efficiency. In contrast, higher drying gas flow resulted in reduced responses. Nebulizing gas flow produced a sharp increase in signal intensity up to approximately 2.0 L/min, after which the response remained stable, suggesting that excessively high flow rates do not further improve ionization. Similar trends were observed for the compounds analyzed in negative ionization mode. Heat block and desolvation line temperatures gradually improved signal intensity, while interface voltage strongly enhanced responses. Conversely, higher drying gas flow slightly reduced the signal, whereas nebulizing gas flow improved the response up to approximately 2.0 L/min, beyond which no further improvement was observed. These results highlight the importance of optimizing ion source parameters to maximize sensitivity and ensure reproducible detection of analytes in both positive and negative ionization modes. The confirmation of all analytes under study in the samples was performed by comparing the ion ratio and their retention times with the data obtained for the respective standards (Table S1). The chromatographic conditions were optimized regarding peak shape, resolution and reproducibility, being described in detail in the Supplementary Materials. The overlaid individual chromatograms of the analyzed compounds in the negative ESI mode using the optimized conditions are shown in Figure 2A. For the compounds analyzed in the positive ESI mode (ginkgolide A, ginkgolide J and andrographolide), a similar optimization study was carried out, with the highest sensitivity and peak shape being obtained when methanol was used as organic eluent together with 0.1% aqueous formic acid, with the mobile phase starting with 10% of methanol. The weak acid was used to improve analyte ionization efficiency and peak shape [35,36]. For the dwell time, the best result in terms of sensitivity and precision was found when 100 ms was used. The overlaid individual chromatograms of the three analyzed compounds in the positive ESI mode are presented in Figure 2B.

To ensure the suitability of the optimized extraction, three spiking levels (50, 75 and 100 mg compound/kg sample) were performed (Table S2, Supplementary Materials). The results of the various assays (Tables S2 and S3, Supplementary Materials) showed high reproducibility, proving the suitability of the selected parameters.

A good linearity of the chromatographic methods was observed over the assayed range, since correlation coefficients (R) higher than 0.998 were obtained for all the studied compounds (Table S4, Supplementary Materials). The LOD and LOQ were determined by the signal-to-noise of the method. For the solid samples, the LOD ranged from 0.00099 (ginkgolide B) to 0.11 mg/kg (quercetin dihydrate), while the LOQ varied between 0.0020 (ginkgolide B) and 0.378 mg/kg (quercetin dihydrate). For liquid samples, the LOD was between 0.05 (ginkgolide A) and 0.3 mg/L (bilobalide), whereas the LOQ was within 0.10 (ginkgolide A) and 1.00 mg/L (bilobalide). In the case of lyophilized samples, the LOD ranged from 0.05 (kaempferol) and 5.98 mg/kg (isorhamnetin) and the LOQ was from 0.20 (kaempferol) and 19.9 mg/kg (isorhamnetin).

#### 3.2.2. Optimization of the Extraction Method and Validation Tests

The extraction of the target compounds is a critical step that could affect the results of the analysis [37]; therefore, several extraction conditions were tested (Section 2.4.2) by adapting the method of Ding et al. [17] who extracted terpene lactones and flavonoids from ginkgo PFS using sonication with methanol. Accordingly, in the present work, different volumes of methanol as extraction solvent, as well as distinct sonication times, were tested and the detailed assays are described in the Supplementary Materials.

Two concentrations of the standard mixture (100 and 1000 µg/L) were injected at the beginning and end of the sequence and the method precision was satisfactory (Table S5). Intra-day precision ranged from 0.16% (bilobalide) to 4.75% (kaempferol) for the standards with 100 µg/L and from 0.42% (ginkgolide B) to 3.40% (ginkgolide J) for the mixture with the higher concentration. For inter-day precision, the RSD varied from 1.33% (ginkgolide J) to 7.35% (quercetin dihydrate) and from 2.51 (ginkgolide B) to 8.75% (quercetin dihydrate) for the standards with the lower and higher concentration, respectively.

#### 3.2.3. Analysis of Ginkgo Leaf Extracts

**Figure 3:**
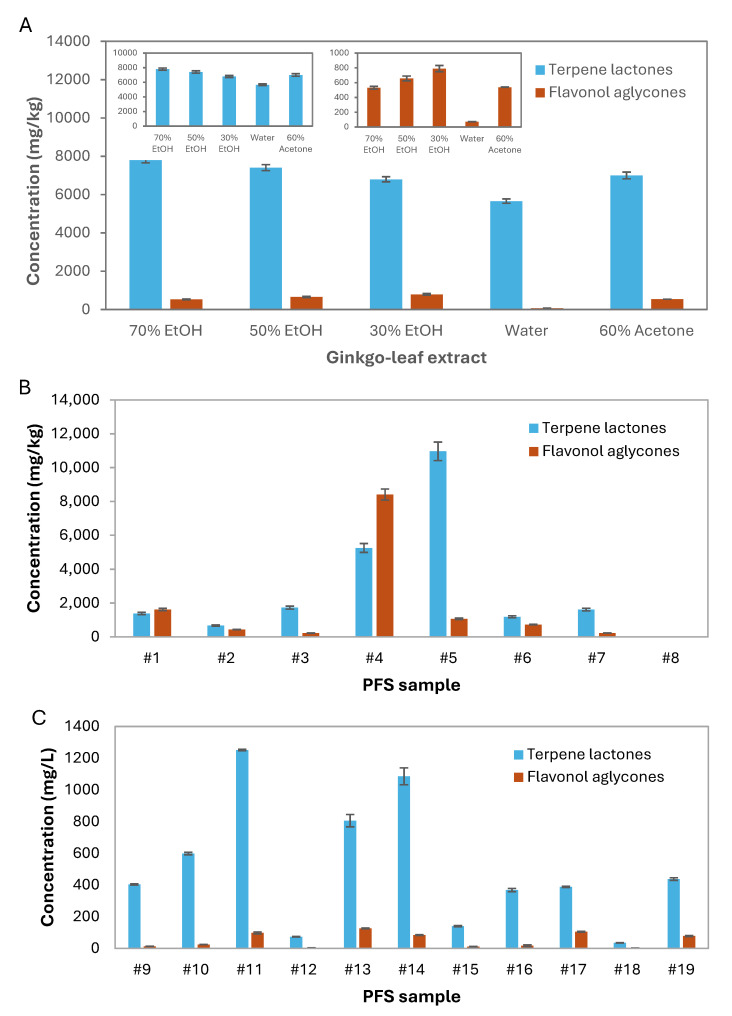
Total contents of terpene lactones and flavonol aglycones analyzed by UHPLC-MS/MS obtained from dried ginkgo leaf extracts using different solvents (30, 50 and 70% of ethanol/water (EtOH), water and 60% of acetone/water) (different letters for the same solvent extraction mean statistically significant differences among the extraction method (*p* < 0.05), following ordinary one-way ANOVA) (**A**), solid (**B**) and liquid (**C**) PFS samples. Values are triplicates (mean ± SD) from 3 independent runs per system.

Dried ginkgo leaf extracts obtained with the different solvents as described in Section 2.2 were analyzed by the developed UHPLC-MS/MS method and the results are summarized in Table 2. The extracts of ethanol/water (70%, *v/v*) showed the highest content of terpene lactones, mainly due to bilobalide, ginkgolide A and ginkgolide B (Figure 3A, Table 2). Such content decreased significantly with the reduction in ethanol concentration until 30%, showing similar values to the 60% acetone extract. In opposition, the water extract had the lowest content of terpene lactones, statistically differing from all the others. In what concerns flavonol aglycones, an opposite trend can be observed since their content increase with the decreasing ethanol concentration, with similar levels being observed for 70% ethanol and 60% acetone extracts (Figure 3A). As expected, water provided the lowest content of flavonol aglycones. Overall, due to the nature of the molecules, higher yields of terpene lactones and flavonol aglycones were obtained with hydroalcoholic and acetone solvents, contrarily to DNA that was higher in the water extract (Figure 1).

#### 3.2.4. Analysis of PFS

The developed UHPLC-MS/MS method was further used to identify and determine the contents of terpene lactones and flavonol aglycones of the PFS samples previously analyzed by real-time PCR. The samples included 8 solid (#1–#8) and 11 liquid formulations (#12–#19) and the results are presented in Table 3. The solid samples showed highly variable contents of both groups of compounds (Figure 3B), possibly due to their diversified formulations, containing either dried-leaf material and/or ginkgo extracts. In addition, it is widely known that several factors, such as climate, soil, and region, among others, can affect the natural chemical composition of plants. Song et al. [24] used HPLC-ESI-MS/MS to evaluate fifteen batches of authentic *G. biloba* leaves collected in the same month from 15 different habitats in China. The samples showed fingerprint chromatograms that were tentatively as being 15 flavonol glycosides, 4 terpene trilactones, 3 flavonol aglycones and 3 biflavones. Despite not being quantified, 3 samples evidenced much lower contents of terpene lactones as compared with the remaining. Based on the calculation of correlation coefficients to estimate the similarity of the chromatographic fingerprints the authors observed variations among samples, which were attributed to different growing conditions. The age and sex of the *G. biloba* trees, not accounted in the reported study, could also be responsible for the variability. Finally, the extraction method, as demonstrated with the application of varied extraction solvents (Table 2), can also affect the final composition of the plant extract.

In solid samples, total terpene lactones varied from 1.7 mg/kg in sample #8 to 10,964.7 mg/kg in sample #5, while total flavonol aglycones ranged from 1.3 mg/kg in sample #8 to 8407.9 mg/kg in sample #4. Except for sample #8, all the compounds under study were identified in the remaining solid PFS. Sample #8 contained only bilobalide, ginkgolides A and J, and quercetin at trace levels (≤1.34 mg/kg). Although labeling indicates a lower content of ginkgo extract as compared to other solid samples, the absence of some terpene lactones and the trace amounts of the others suggest the partial substitution of ginkgo in sample #8. On the other hand, samples #1 and #4 revealed the highest contents of flavonol aglycones (Figure 3B) mainly due to the huge quercetin levels (Table 3), suggesting the addition of the compound from other sources than from ginkgo plant material. The possible adulteration by the addition of aglycones has been previously reported by Wohlmuth et al. [4] who found very high levels of quercetin and kaempferol in *G. biloba* retail products from Australia (one tablet and two capsules). The suspicion of adulteration by aglycones addition was also reported by Czigle et al. [7] who found high levels of quercetin and no appreciable amounts of terpene lactones in one of the eleven samples evaluated, while the other 2 samples showed *G. biloba* in levels lower than the declared together with high amounts of flavonol aglycones.

Regarding the liquid PFS samples (#9–#19), the labeled information is even scarcer than in solid ones since only some samples declared the amount/proportion of ginkgo leaves, without any mention to the content of flavonoids and/or terpene lactones. In these samples, variable levels of total terpene lactones were found, with contents ranging from 35 mg/L (sample #18) to 1251 mg/L (sample 11#) (Table 3, Figure 3C). The difference among the samples is notable, suggesting distinct levels of quality and/or types of preparations. Samples #9–#11, described as water/glycerin extracts, and samples #12–#19, corresponding to hydroalcoholic extracts prepared with varied proportions of ethanol, did not present any pattern relating to the concentration of compounds with the type of liquid formulation. In comparison with the solid PFS, the liquid samples showed much lower contents of the two groups of compounds, with samples #12 and #18 revealing minute/negligible amounts of compounds, while declaring around 10% of the plant used for preparation (Table 1).

Concerning the labeled groups of compounds, only 5 samples declare phytochemical contents (#1, #2, #4, #5 and #11), which, according to the relevant data (Table 1), were converted to mg per kg or mg per liter for comparative purposes with the herein determined levels of compounds. Table 4 shows the comparison of the declared and estimated contents of terpene lactones and flavonoids/flavonol aglycones for these samples. Samples #1 and #5 contain standardized extracts, referring the contents of both groups of compounds (terpene lactones and flavonoids). While the content of terpene lactones estimated for sample #5 was similar to that declared on the label, in sample #1, the same content was approximately 5-fold lower than the declared. This result suggests that, besides the probable adulteration by the addition of quercetin, sample #1 is also non-compliant due to the lower content of ginkgo. With respect to flavonoids, they were about 20- and 50-fold lower than the labeled values for samples #1 and #5, respectively, and about 20-fold lower for the other samples (#2, #4 and #11) that only declared contents of flavonoids (Table 4). However, this difference is expected and justified as ginkgo naturally contains low levels of aglycones since flavonoids are found mainly in their glycoside form [3,38,39].

### 3.3. Discussion

Generally, chemical approaches have been the most applied and recommended by authorities to assess the authenticity, quality and safety of foods. However, over the past few decades, DNA-based methods have emerged as powerful alternative tools to assess the botanical origin of food and herbal products. Chemical methods can detect the addition of synthetic compounds but can fail in the accurate identification of the botanical species due to the sample complexity and natural variability associated with phytochemical compounds. Conversely, DNA-based methods can identify the species of origin, nonetheless, they are incapable of detecting the addition of synthetic compounds. Therefore, the applicability of DNA and chemical approaches to analyze ginkgo leaf extracts and samples of PFS in diversified formulations was herein demonstrated to evaluate the potential of using both techniques as complementary approaches for botanical authentication. Given that different extraction processes can be applied to produce ginkgo extracts and this information is generally not disclosed in commercially available PFS, in this study, in-house dried ginkgo leaf extracts were also prepared using different solvents with two main goals: assessing ginkgo DNA traceability from distinct formulations and providing a comprehensive profile of the two major compound groups, namely terpene lactones and flavonol aglycones.

Considering the obtained in-house ginkgo leaf extracts, the ethanol/water solvents provided the highest recoveries of phytochemicals, while water extracts, as expected, yielded the highest DNA quantities. Nevertheless, it was possible to trace ginkgo DNA in all types of extracts. Interestingly, despite the expected DNA precipitation with ethanol [36] it was still possible to obtain amplifiable DNA with all tested ethanol proportions. The extraction with 60% acetone/water revealed the poorest DNA amplification but yielded intermediate recoveries of phytochemical compounds. In opposition, the extraction with water yielded the highest DNA levels, though the lowest phytochemical contents. These results align with the higher affinity of phytochemical compounds for organic solvents, in opposition to DNA molecules [40,41]. Consequently, PFS containing ginkgo extracts produced with acetone are likely to contain only trace amounts of ginkgo DNA, being more prone to unsuccessful DNA extraction. Despite the observed significant differences in the phytochemical contents of ginkgo leaf extracts (Figure 3A), the total amounts varied between 7536.7 mg/kg for 60% acetone and 8335.4 mg/kg for 30% ethanol, except for water that yielded the lowest content (5730.1 mg/kg). As mentioned, to account for possible differences associated with the extraction process, the same preparation of ginkgo leaves (prepared as a composite mixture of different authenticated ginkgo leaves from botanical gardens) was submitted to extraction with different solvents. With this procedure, the variability in phytochemical composition due to different factors, such as climate, soil, light and genetics, could be neglected. In this study, comparisons were made only considering the general profile of the two main groups of ginkgo bioactive compounds. Figure 3A shows that irrespective of the solvent used for extraction, a pattern is observed with all extracts showing a much higher concentration of terpene lactones as compared to the amount of flavonoid aglycones.

Concerning commercial PFS, in general, the solid forms presented higher amounts of bioactive compounds as compared to the liquid ones (Table 3). The results of DNA analysis corroborated this finding since, in general, a higher content of ginkgo DNA was also obtained for the solid PFS (Table 1). Regarding the phytochemical compounds, it is interesting to analyze and compare the profiles of terpene lactones and flavonol aglycones of the analyzed samples with those of in-house ginkgo leaf extracts. In solid PFS, only samples #3, #5 and #7 exhibit ratios comparable profiles with the in-house extracts, while all the others, particularly samples #1 and #4 show distinct profiles due to the relatively higher contents of flavonol aglycones (Table 3). In both samples, this was mainly due to the high quercetin content, therefore reinforcing adulteration by the addition of this compound. In these cases, as for most solid samples, the variable DNA content is not very informative regarding potential adulterations. However, in sample #8, the vestigial amounts of phytochemicals, corroborating the low ginkgo DNA content, suggest the reduction or substitution of ginkgo extract (Table 1 and Table 3).

The scenario of liquid samples is rather different since most of them show profiles of terpene lactones and flavonol aglycones comparable to the in-house ginkgo leaf extracts, suggesting closely related patterns (Table 3). In addition, it can be observed that sample #18 was one of the few that did not evidence bilobalide as a major terpene lactone, presenting instead higher contents of ginkgolides A, B and C. According to authors who studied the contents of terpene lactones in different parts of the ginkgo tree, the leaves are generally relatively richer in bilobalide, while ginkgolides are frequently found in higher amounts in the roots and stems [42,43]. Therefore, the use of other parts of the ginkgo plant than the leaves would explain both the lower content of bilobalide and the relatively high amount of ginkgo DNA, which is in fact the liquid sample with the highest DNA content.

The results of Table 1 show that three PFS samples did not provide detectable ginkgo DNA, namely a solid (#3) and two liquids (#10 and #16). They showed reasonable amounts of all bioactive compounds in the UHPLC-MS/MS analysis, which indicates the presence of ginkgo, though unsuccessful DNA detection. Besides the choice of the extraction solvent, the inefficient DNA recovery may be due to its adsorption to some excipient present in the specific formulation of those products [25] emphasizing the need of complementarity analytical approaches in these cases. In opposition, the solid samples #1, #5 and #7 yielded the highest levels of amplifiable DNA (Table 1), much higher than the ginkgo leaf extract with water (Figure 1B), having moderate to high amounts of phytochemicals. Such high DNA yields may be due to the addition of plant material (claimed in samples #1 and #7) as they contain more than 10-fold higher DNA than the ginkgo leaf extract for the best performing solvent for DNA (110.69 pg for water). This is a notable finding regarding the usefulness of DNA analysis for evaluating the compliance of labeled information of PFS.

Generally, phytochemical composition suggests that solid samples are mostly not according to the pattern of ginkgo leaf extracts, while liquid samples seem to follow their trend. Moreover, phytochemical analysis can detect the addition of compounds and the enrichment of root/stem material rather than ginkgo leaves. On the other hand, the detection of ginkgo DNA provides the unequivocal presence of ginkgo material, which at high amounts (>100 pg) may confirm the presence of plant material, thus differentiating the addition of ginkgo extracts from ginkgo-plant material. However, the cases of non-detectable DNA should be complemented with phytochemical analysis as its low recovery may be due to the use of leaf extracts produced with acetone or ethanol rich solvents and/or the use of DNA adsorbing excipients.

## 4. Conclusions

In the present work, DNA and phytochemical markers were used to identify and authenticate the botanical origin of ginkgo leaf extracts and PFS. The combination of the proposed approaches was complementary in identifying and detecting different adulteration procedures. While DNA-based approaches can unequivocally detect the presence/absence of botanical species, the chemical methods unveil the contents of bioactive compounds and the addition of synthetic compounds. Effectively, both systems contributed to identifying possible authenticity issues in commercial samples. The combination of results suggests that 4 out of 19 samples were adulterated, two samples by the addition of quercetin as established by the developed UPLC-MS/MS approach, while the other two samples indicate a reduced amount of ginkgo phytochemicals, which was corroborated by low DNA content in one sample, while the other suggest the use of non-leaf ginkgo material.

Hence, both DNA and chemical approaches proved to be efficient, accurate and robust tools, being suitable for the complementary analysis by regulatory authorities and control laboratories to assess the authenticity of PFS.

## Figures and Tables

**Table 1:** Commercial samples of PFS and results of qualitative PCR and real-time PCR targeting the ITS1 region of ginkgo.

Code	Commercial Samples	Relevant Label Information	Qualitative PCR	Cq (Mean ± SD ^1^)	Ginkgo DNA (pg) (Mean ± SD ^1^)
#1	*Ginkgo biloba* capsules	*G. biloba* leaves (80%) and dry extract 50:1 (24% flavonol glycosides, 6% terpene lactones) (12%) (28.8 mg flavonol glycosides, 7.2 mg terpene lactones per 2 capsules, 920 mg)	+	23.56 ± 0.21	3714.5 ± 479.2
#2	Ginkoro tablets	*G. biloba* leaves 400 mg (0.5% flavonoids), *G. biloba* dry extract 20 mg (24% flavonoids) per 2 capsules (660 mg)	+	27.64 ± 0.36	220.1 ± 33.1
#3	Cerebrum + ginkgo capsules	*G. biloba* (11 mg) per capsule (495 mg).	ND ^2^	ND	
#4	*Ginkgo biloba* capsules	*G. biloba* dry extract (24% flavonoids). Capsule of 461 mg.	+	29.37 ± 0.35	66.24 ± 10.70
#5	*Ginkgo biloba* tablets	*G. biloba* dry extract (100 mg); flavonol glycosides (24 mg); terpene lactones (6 mg) per capsule (433 mg).	+	24.69 ± 0.16	1691.2 ± 189.1
#6	Ginseng + Ginkgo + Gotu kola capsules (pure herb)	*G. biloba* (130 mg/capsule of 450 mg)	+	30.39 ± 0.46	33.26 ± 8.93
#7	*Ginkgo biloba* capsules (pure herb)	*G. biloba* (500 mg/capsule)	+	22.84 ± 0.21	6107.4 ± 654.5
#8	Ginseng and *Ginkgo biloba* tablets	*G. biloba* extract (60 mg)	±	32.06 ± 0.39	10.42 ± 2.65
#9	Non-alcoholic liquid extract of *Ginkgo biloba*	*G. biloba* leaf extract (125 mg) (water/glycerine)	±	38.14 ± 1.21	0.22 ± 0.17
#10	Liquid extract of *Ginkgo biloba*	*G. biloba* leaf extract (250 mg) (water/glycerine)	±	38.82 ± 0.95	<LOD ^3^
#11	Liquid extract of *Ginkgo biloba*	*G. biloba* L. leaves (lyophilised extract, 120 mg/dose) (water/glycerine). Flavonoids: 6 mg/3 mL.	+	36.50 ± 0.99	0.72 ± 0.21
#12	Hydroalcoholic extract of ginkgo	*G. biloba* leaf + button extract 10% (330 mg/dose)	+	37.58 ± 0.67	0.30 ± 0.12
#13	Hydroalcoholic extract of ginkgo	*G. biloba* leaf extract (30% alcohol)	+	36.69 ± 0.88	0.27 ± 0.09
#14	Hydroalcoholic extract of ginkgo	*G. biloba* extract	+	36.87 ± 0.99	0.35 ± 0.10
#15	Hydroalcoholic extract of ginkgo	*G. biloba* young leaves (5% plant) (25% ethanol)	+	34.11 ± 0.36	1.69 ± 0.42
#16	Hydroalcoholic extract of ginkgo	*G. biloba* L. leaves (45% alcohol)	ND	ND	
#17	Hydroalcoholic extract of ginkgo	*G. biloba* fresh plant (65% alcohol)	+	34.83 ± 0.38	1.54 ± 0.46
#18	Hydroalcoholic extract of ginkgo	*G. biloba* (10.3% plant) (32% alcohol)	+	33.14 ± 0.19	4.65 ± 0.58
#19	Hydroalcoholic extract of ginkgo	*G. biloba* leaves (65% alcohol)	+	35.62 ± 0.33	0.88 ± 0.17

^1^ Mean values (cycle of quantification (Cq) and estimated ginkgo DNA $\left(DNA(ng)=10^{\left(\frac{C_{q}-b}{m}\right)}\right)$ ± standard deviation (at least n = 4 replicates of 2 independent assays). ^2^ ND, not detected. ^3^ LOD, limit of detection.

**Table 2:** Phytochemical compounds of dried ginkgo leaf extracts using different solvents were analyzed by UHPLC-MS/MS.

Leaf Extracts	(-)-Bilobalide	Ginkgolide A	Ginkgolide B	Ginkgolide C	Ginkgolide J	Isorhamnetin	Kaempferol	Quercetin Dihydrate
Concentration (mg/kg) ± RSD (%)								
70% EtOH	2819.99 ± 6.28	1992.02 ± 5.78	1135.42 ± 7.72	1194.03 ± 6.99	663.28 ± 2.18	229.10 ± 0.53	86.16 ± 6.75	215.37 ± 7.17
50% EtOH	2898.02 ± 1.75	1488.69 ± 1.93	993.60 ± 7.60	1246.72 ± 7.97	777.24 ± 0.53	294.27 ± 7.39	127.13 ± 6.06	235.03 ± 1.81
30% EtOH	3079.95 ± 6.93	1215.09 ± 1.57	762.58 ± 5.42	1073.11 ± 3.12	665.13 ± 2.85	370.49 ± 7.44	163.40 ± 6.11	255.59 ± 6.26
Water	2241.63 ± 1.96	811.25 ± 3.06	806.44 ± 2.93	1164.17 ± 4.96	634.96 ± 2.39	33.48 ± 1.46	4.93 ± 7.01	33.22 ± 1.59
60% Acetone	2762.67 ± 2.50	1098.65 ± 1.37	1047.36 ± 1.43	1285.56 ± 2.08	756.23 ± 2.17	286.04 ± 1.80	126.07 ± 6.03	217.47 ± 4.29
LOD (mg/kg)	3.75	0.47	0.50	0.30	2.50	5.98	0.05	1.07
LOQ (mg/kg)	12.75	1.56	1.50	1.00	8.50	19.92	0.20	3.55

**Table 3:** Phytochemical compounds of solid and liquid plant food supplements (PFS) analyzed by UHPLC-MS/MS.

Sample	(-)-Bilobalide	Ginkgolide A	Ginkgolide B	Ginkgolide C	Ginkgolide J	Isorhamnetin	Kaempferol	Quercetin Dihydrate
Solid PFS-Concentration (mg/kg) ± RSD ^1^ (%)								
#1	561.09 ± 2.21	332.97 ± 7.14	146.85 ± 2.63	202.81 ± 3.18	137.10 ± 4.06	188.08 ± 5.96	31.93 ± 4.10	1399.22 ± 1.48
#2	149.91 ± 0.94	136.14 ± 2.67	145.30 ± 3.03	174.91 ± 3.36	62.46 ± 8.07	92.35 ± 5.00	42.11 ± 5.98	295.61 ± 10.20
#3	482.83 ± 6.62	349.60 ± 8.54	366.34 ± 2.26	364.11 ± 4.40	168.40 ± 3.47	33.72 ± 6.35	30.71 ± 9.84	159.62 ± 11.5
#4	2359.69 ± 3.99	616.92 ± 3.69	246.37 ± 4.80	1471.26 ± 2.81	550.86 ± 5.78	803.97 ± 3.76	326.22 ± 2.12	7277.71 ± 1.82
#5	4387.90 ± 6.32	1845.86 ± 9.14	1363.47 ± 6.62	2470.34 ± 5.64	897.12 ± 5.44	218.08 ± 8.10	186.92 ± 6.61	665.62 ± 10.8
#6	291.29 ± 3.42	274.57 ± 8.49	212.44 ± 2.28	251.94 ± 2.69	158.63 ± 3.02	209.11 ± 4.40	237.61 ± 2.51	277.80 ± 2.97
#7	442.25 ± 1.63	375.3 ± 3.17	332.20 ± 1.78	309.42 ± 1.71	156.37 ± 2.82	78.46 ± 4.19	40.71 ± 2.13	112.59 ± 7.62
#8	0.300 ± 20.5	0.980 ± 13.4	<LOD	<LOD	0.380 ± 12.8	<LOD	<LOD	1.34 ± 8.12
LOD ^2^ (mg/kg)	0.003	0.001	0.001	0.001	0.14	0.047	0.002	0.11
LOQ ^3^ (mg/kg)	0.008	0.002	0.002	0.003	0.458	0.157	0.005	0.378
Liquid PFS-Concentration (mg/L) ± RSD (%)								
#9	122.85 ± 1.34	158.30 ± 1.05	39.46 ± 4.11	67.90 ± 5.78	14.87 ± 3.46	5.35 ± 8.15	2.99 ± 5.87	4.76 ± 5.40
#10	256.59 ± 4.13	143.40 ± 4.72	62.91 ± 4.16	101.20 ± 1.84	34.28 ± 1.94	10.62 ± 4.04	4.95 ± 8.42	9.06 ± 2.97
#11	257.33 ± 2.29	311.62 ± 6.23	408.03 ± 1.25	231.53 ± 6.03	42.44 ± 2.41	1.47 ± 7.10	30.86 ± 2.08	65.37 ± 8.10
#12	39.38 ± 5.42	9.77 ± 1.25	7.34 ± 3.50	11.39 ± 3.30	5.40 ± 0.43	1.74 ± 8.57	1.08 ± 5.27	1.48 ± 4.29
#13	376.90 ± 6.65	128.69 ± 1.38	93.97 ± 6.47	142.11 ± 5.16	64.11 ± 1.41	33.53 ± 4.12	36.00 ± 7.27	56.70 ± 2.62
#14	475.84 ± 5.98	193.23 ± 2.61	121.45 ± 8.12	214.89 ± 7.47	79.95 ± 1.07	27.53 ± 2.05	17.04 ± 7.62	38.99 ± 4.12
#15	57.77 ± 3.35	20.18 ± 1.31	19.62 ± 2.91	34.52 ± 5.09	7.45 ± 3.69	3.78 ± 4.76	3.72 ± 6.71	3.85 ± 0.41
#16	93.59 ± 1.60	101.47 ± 5.94	66.06 ± 1.47	72.22 ± 3.24	34.66 ± 3.76	3.43 ± 4.37	4.27 ± 5.45	9.30 ± 5.08
#17	157.32 ± 4.66	63.69 ± 1.53	56.53 ± 6.22	66.97 ± 1.73	43.24 ± 1.44	37.35 ± 3.59	32.99 ± 3.83	35.10 ± 6.45
#18	1.91 ± 2.45	5.79 ± 1.37	12.83 ± 2.68	13.78 ± 2.15	0.74 ± 4.55	1.91 ± 2.88	0.63 ± 4.81	0.33 ± 5.76
#19	211.29 ± 3.11	30.07 ± 1.99	37.29 ± 8.51	73.05 ± 2.17	85.62 ± 4.18	26.39 ± 1.91	16.63 ± 6.54	35.01 ± 5.91
LOD (mg/L)	0.30	0.05	0.10	0.06	0.13	0.30	0.08	0.27
LOQ (mg/L)	1.00	0.10	0.38	0.18	0.43	0.99	0.26	0.91

^1^ RSD, relative standard deviation; ^2^ LOD, limit of detection; ^3^ LOQ, limit of quantification.

**Table 4:** Comparison of the estimated contents of phytochemical compounds of PFS samples by UHPLC-MS/MS and the labeled information.

Sample	Labeled		Estimated by UHPLC-MS/MS	
	Terpene Lactones	Flavonol Glycosides ^1^/ Flavonoids ^2^	Terpene Lactones	Flavonol Aglycones
	Concentration (mg/kg)			
#1	7826.1	31,304.3 ^1^	1380.8	1619.2
#2		33,333.3 ^2^	668.7	430.1
#4		240,000.0 ^2^	5245.1	8407.9
#5	13,856.8	55,427.3 ^1^	10,964.7	1070.6
	Concentration (mg/L)			
#11		2000 ^2^	1251.0	97.7

Calculated concentration based on the label information: ^1^ Flavonol glycosides and ^2^ Flavonoids.

## Data Availability

The raw data supporting the conclusions of this article will be made available by the authors on request.

## Supplementary Materials

The following supporting information can be downloaded at: https://www.mdpi.com/article/10.3390/foods14173111/s1. (1) Optimization of chromatography conditions; (2) Optimization of the extraction method and evaluation of matrix effect; Tables S1–S5; Figures S1–S5. Table S1. Optimized mass spectrometry conditions, retention time and ion ratio for the selected compounds analyzed in the positive and negative electrospray ionization (ESI) modes. Table S2. Recoveries of the studied compounds at three fortification levels for bilobalide, ginkgolides A, B, C and J, isorhamnetin, kaempferol and quercetin dihydrate. Table S3. Average recoveries and relative standard deviations (RSD, %) at the three fortification levels of each target compound in the study. Table S4. Calibration curve equation, coefficient of determination (R²), and correlation coefficient (R) for both MRM transition and for each compound. Table S5. Intra- and inter-day precision at two concentration levels (100 and 1000 µg/L) for bilobalide, ginkgolides A, B, C, and J, isorhamnetin, kaempferol and quercetin dihydrate. Figure S1. Amplification curves (A) and respective calibration curve (B) of a real-time PCR assay with a hydrolysis probe targeting ITS1 region of G. biloba using 10-fold serially diluted ginkgo DNA (20 ng to 0.2 pg) (n = 3 replicates). Cq, cycle of quantification. Figure S2. Areas obtained in the tests performed in the positive ionization mode for achieving the highest sensitivity, varying the heat block temperature, desolvation line temperature, interface voltage, drying gas flow, and nebulizing gas flow parameters for each compound and the sum area obtained for each ion source parameter. Figure S3. Areas obtained in the tests performed in the negative ionization mode for achieving the highest sensitivity, varying the heat block temperature, desolvation line temperature, interface voltage, drying gas flow, and nebulizing gas flow parameters for each compound and the sum area obtained for each ion source parameter. Figure S4. Recovery rates and RSD (n=2) obtained by testing different extraction times using an ultrasonic processor. Figure S5. Matrix effect (ion suppression/ion enhancement) for solid and liquid PFS and lyophilized plant extracts without dilution and with various dilution factors (2×, 5×, 10×, 25×, 50×) for the studied compounds.
