# Supplementary material for: Towards Botanical Authentication of Ginkgo Food Supplements: A Holistic Approach Based on Phytochemical and Genomic Markers
Source: Foods. 2025 Sep 5;14(17):3111. doi: 10.3390/foods14173111 (PMC12428234; doi:10.3390/foods14173111)
Supplement: Supplementary file 1 [file foods-14-03111-s001.zip › foods-3826091-supplementary.pdf]

Supplementary material

Towards botanical authentication of ginkgo food supplements: A holistic approach based on phytochemical and genomic markers

Liliana Grazina[1§], Paula Paíga[2§], Joana S. Amaral[3,*], Joana Costa[1], Manuela M. Moreira[2], Cristina Delerue-Matos[2], Isabel Mafra[1,*]

[1] REQUIMTE/LAQV, Faculdade de Farmácia, Universidade do Porto, Rua de Jorge Viterbo Ferreira, 228, 4050-313 Porto, Portugal

[2] REQUIMTE/LAQV, Instituto Superior de Engenharia do Porto, Instituto Politécnico do Porto, Rua Dr. António Bernardino de Almeida, 431, 4249-015 Porto, Portugal

[3] CIMO, LA SusTEC, Instituto Politécnico de Bragança, Campus de Santa Apolónia, 5300-253 Bragança, Portugal

[§]The authors contributed equally to the present work.

[*] Corresponding author. Tel.: +351 220428500

E-mail address: isabel.mafra@ff.up.pt (I. Mafra), jamaral@ipb.pt (J. Amaral)

1. Optimization of chromatography conditions

The chromatographic conditions were optimized to enhance peak shape, resolution, and reproducibility. Hence, several variables were evaluated for method optimization, including the eluents (acetonitrile and methanol for the organic phase, and ultra-pure water and acidified water with formic or acetic acids in different concentrations for the aqueous phase; the initial quantity of the organic phase in the eluent was tested from 5 to 50%), elution mode (isocratic or gradient elution with different ramp programs), oven temperature (30 and 40 ºC) and dwell time (1, 5, 10, 15, 25, 50, 55, 60, 65, 70, 75, 80, 85, 90, 95 and 100 ms). In the negative ionization mode, organic solvents and ultra-pure water are normally used as eluents [29]**.** In this work, the best results were achieved with methanol, which was therefore selected as the organic phase. Thus, several proportions of methanol in ultra-pure water were tested, with 5% of methanol in the initial composition of the mobile phase with a linear gradient to 100% in 3 min, revealing the best conditions for the sensitivity when compounds were analyzed in the negative ESI mode. Nevertheless, in these conditions, four compounds showed good peak shape ((-)-bilobalide, ginkgolide C, ginkgolide B and andrographolide), while others (isorhamnetin,

kaempferol, and quercetin dihydrate) exhibited a tailing shape. Since mobile-phase additives can be used to improve analyte ionization efficiency and peak shape [33] the addition of different proportions of acetic or formic acids in the aqueous mobile phase was tested owing to their frequent use in the chromatographic analysis of plant flavonoids **[3]**. In the present study, the addition of acetic acid to the aqueous phase gave the best results, thus, being assayed in the concentrations of 0.05%, 0.10% and 0.20%. Isorhamnetin, kaempferol and quercetin improved their peak shape, though their response decreased with the increasing acetic acid concentration, leading to the selection of the lowest amount. This finding was consistent with the observations of Wu et al.**[4].** Most of the studied compounds were not affected by the oven temperature increase, except for kaempferol, andrographolide and quercetin dihydrate, which presented area reductions of 21%, 24% and 69%, respectively. Therefore, an oven temperature of 30 ºC was selected for analysis in the negative ESI mode. Finally, the dwell time was also a studied variable, for which 16 tests were performed with triplicate injections. The best compromise for sensitivity and precision was found when 60 ms was used.

2. Optimization of the extraction method and evaluation of matrix effect

Different volumes of methanol (5, 10 and 20 mL), as the extraction solvent, were assayed to dissolve 100 mg of sample. Despite incomplete dissolution, which can be attributed to insoluble excipients used in the PFS formulations, the highest volumes (10 and 20 mL) were more efficient in dissolving the sample than 5 mL of methanol. In addition, as the starting mobile phase has a high percentage of aqueous solvent that can lead to the occurrence of precipitation phenomena [29], a preliminary test was conducted before chromatographic analysis. Hence, 400 µL of ultra-pure water were added to 100 µL of the extracts obtained from the different ratio methanol:sample (5 mL/100 mg, 10 mL/100 mg and 20 mL/100 mg), followed by vortexing and storage overnight at 4 ºC. Except for the extraction volume of 5 mL, both higher volumes resulted in clear solutions. The sample dilution factor was crucial for accurate analysis, so 10 mL of methanol was selected for extraction. Subsequently, different sonication times (5, 10, 15, 30 and 50 min) were evaluated in triplicate regarding the recovery for each target compound. The recoveries were similar among the different extraction times (Fig. S4), but the RSD increased with extraction time, leading to the selection of 5 min of ultrasonic extraction. In the study by Ding et al. [17] longer extraction times (50 min) were required, which can

possibly be explained by the use of an ultrasonic probe in the present study, as it may be more effective than the ultrasonic bath used by Ding et al. [17].

The matrix effect (ME) was evaluated for each type of sample analyzed, including solid and liquid PFS as well as lyophilized plant extracts. Samples were initially analyzed without dilution, followed by analyses at various dilution factors (2x, 5x, 10x, 25x and 50x). Ion suppression was observed in all compounds studied when samples were analyzed without dilution. As shown in Figure S5, the ME decreased progressively with increasing dilution, becoming less pronounced at higher dilution factors, which is consistent with expectations.

17. Ding, S., Dudley, E., Plummer, S., Tang, J., Newton, R. P., & Brenton, A. G. Fingerprint profile of *Ginkgo biloba* nutritional supplements by LC/ESI-MS/MS. *Phytochem.* **2008**, *69*(7), 1555-1564. DOI: 10.1016/j.phytochem.2008.01.026

28. Spréa, R. M., Caleja, C., Pinela, J., Finimundy, T. C., Calhelha, R. C., Kostić, M., Sokovic, M., Prieto, M. A., Pereira, E., Amaral, J. S., & Barros, L. Comparative study on the phenolic composition and in vitro bioactivity of medicinal and aromatic plants from the Lamiaceae family. *Food Res. Int.* **2022**, *161*, 111875. DOI: 10.1016/j.foodres.2022.111875

29. Paíga, P., Rodrigues, M. J. E., Correia, M., Amaral, J. S., Oliveira, M. B. P. P., & Delerue-Matos, C. Analysis of pharmaceutical adulterants in plant food supplements by UHPLC-MS/MS. *Eur. J. Pharmac. Sci.* **2017**, *99*, 219-227. DOI: 10.1016/j.ejps.2016.12.024

35. Monnin, C., Ramrup, P., Daigle-Young, C., Vuckovic, D. Improving negative liquid chromatography/electrospray ionization mass spectrometry lipidomic analysis of human plasma using acetic acid as a mobile-phase additive. *Rapid Commun. Mass Spectrom.* **2018**, *32*(3), 201-211. DOI: 10.1002/rcm.8024

36. Wu, Z., Gao, W., Phelps, M. A., Wu, D., Miller, D. D., Dalton, J. T. Favorable effects of weak acids on negative-ion electrospray ionization mass spectrometry. *Anal. Chem.* **2004**, *76*(3), 839-847. DOI: 10.1021/ac0351670

Table S1. Optimized mass spectrometry conditions, retention time and ion ratio for the selected compounds analyzed in the positive and negative electrospray ionization (ESI) modes.

Compounds*	ESI	Precursor ion (m/z)	Product 1				Product 2				Retention time (min)			Ion Ratio		
			m/z	Q1 Pre-Bias (V)	CE	Q3 Pre-Bias (V)	m/z	Q1 Pre-Bias (V)	CE	Q3 Pre-Bias (V)	Average (min)	RSD (%)	n	Average	RSD (%)	n
**(-)-Bilobalide**	-	325.00	163.15	24	21	16	251.15	22	11	15	3.272	0.04	45	3.75	2.21	30
**Ginkgolide C**	-	439.05	383.20	18	17	20	125.00	29	33	19	3.321	0.05	45	2.05	3.07	28
**Ginkgolide B**	-	423.05	367.25	30	17	12	125.10	28	31	12	3.582	0.05	45	2.09	2.21	28
**Andrographolide**	-	331.15	303.30	25	25	22	-	-	-	-	3.776	0.04	45	-	-	-
**Quercetin**	-	301.00	151.05	21	23	15	179.05	21	19	12	3.838	0.12	45	2.42	5.63	24
**Kaempferol**	-	285.00	117.00	21	46	19	143.05	19	34	14	3.973	0.06	45	1.19	3.44	22
**Isorhamnetin**	-	314.95	300.00	25	22	15	151.05	33	31	15	3.992	0.05	45	4.08	3.66	22
**Ginkgolide J**	+	446.95	23.00	-23	-37	-25	-	-	-	-	3.133	0.11	45	-	-	-
**Ginkgolide A**	+	426.10	409.10	-22	-20	-29	18.05	-30	-46	-20	3.422	0.09	45	7.31	1.91	24
**Andrographolide**	+	405.05	373.05	-26	-8	-27	-	-	-	-	3.678	0.10	45	-	-	-

* The compounds were organized by their retention times in each ionization mode.

Table S2. Recoveries of the studied compounds at three fortification levels for bilobalide, ginkgolides A, B, C and J, isorhamnetin, kaempferol and quercetin dihydrate.

Compounds	Spiking Level I-100 mg/kg			
	Recovery 1 (%)	Recovery 2 (%)	Average Recovery (%)	RSD (%)
**Bilobalide**	110.2	106.4	**108.3**	2.48
**Ginkgolide A**	91.48	90.39	**90.93**	0.848
**Ginkgolide B**	106.7	107.5	**107.1**	0.515
**Ginkgolide C**	107.9	109.9	**108.9**	1.30
**Ginkgolide J**	100.5	100.3	**100.4**	0.143
**Isorhamnetin**	98.36	103.2	**100.8**	3.36
**Kaempferol**	101.4	103.4	**102.4**	1.39
**Quercetin dihydrate**	105.3	112.0	**108.6**	4.37

Compounds	Spiking Level II-75 mg/kg			
	Recovery 1 (%)	Recovery 2 (%)	Average Recovery (%)	RSD (%)
**Bilobalide**	97.72	108.4	**103.1**	7.32
**Ginkgolide A**	96.11	95.11	**95.61**	0.739
**Ginkgolide B**	103.2	105.0	**104.1**	1.18
**Ginkgolide C**	97.84	109.6	**103.7**	8.01
**Ginkgolide J**	94.53	98.98	**96.75**	3.26
**Isorhamnetin**	90.89	89.05	**89.97**	1.45
**Kaempferol**	85.03	93.72	**89.38**	6.87
**Quercetin dihydrate**	91.01	94.06	**92.53**	2.33

Compounds	Spiking Level III-50 mg/kg			
	Recovery 1 (%)	Recovery 2 (%)	Average Recovery (%)	RSD (%)
**Bilobalide**	91.83	89.97	**90.90**	1.44
**Ginkgolide A**	95.56	91.02	**93.29**	3.44
**Ginkgolide B**	98.86	92.38	**95.62**	4.80
**Ginkgolide C**	90.40	84.66	**87.53**	4.63
**Ginkgolide J**	97.72	104.4	**101.1**	4.70
**Isorhamnetin**	100.4	86.64	**93.54**	10.4
**Kaempferol**	77.03	65.31	**71.17**	11.6
**Quercetin dihydrate**	71.97	86.37	**79.17**	12.9

Table S3. Average recoveries and relative standard deviations (RSD, %) at the three fortification levels of each target compound in the study.

Compounds	Recovery Average (%)	RSD (%)
**Bilobalide**	100.8	8.71
**Ginkgolide A**	93.28	2.76
**Ginkgolide B**	102.3	5.61
**Ginkgolide C**	100.1	10.8
**Ginkgolide J**	99.41	3.30
**Isorhamnetin**	94.76	7.15
**Kaempferol**	87.65	16.9
**Quercetin dihydrate**	93.45	15.2

Table S4. Calibration curve equation, coefficient of determination (R^2), and correlation coefficient (R) for both MRM transition and for each compound.

Compounds	MRM Transition	Regression				
		Calibration curve equation (Y= mX+b)	m	b	R^2	R
**Bilobalide**	MRM1	Y = (103.692)X + (0.0797028)	7.97E-02	1.04E+02	0.99969	0.99985
	MRM2	Y = (30.0110)X + (0.0208681)	2.09E-02	3.00E+01	0.99957	0.99978
**Ginkgolide A**	MRM1	Y = (1.95517)X + (0.00246788)	2.47E-03	1.96	0.99787	0.99893
	MRM2	Y = (0.240252)X + (0.000200070)	2.00E-04	2.40E-01	0.99747	0.99874
**Ginkgolide B**	MRM1	Y = (81.8014)X + (0.0450831)	4.51E-02	8.18E+01	0.99977	0.99988
	MRM2	Y = (39.2758)X + (0.0196087)	1.96E-02	3.93E+01	0.99951	0.99975
**Ginkgolide C**	MRM1	Y = (58.5392)X + (0.0306340)	3.06E-02	5.85E+01	0.99982	0.99991
	MRM2	Y = (29.1596)X + (0.0140890)	1.41E-02	2.92E+01	0.99983	0.99991
**Ginkgolide J**	MRM1	Y = (0.397459)X + (0.000216287)	2.16E-04	3.97E-01	0.99867	0.99934
**Isorhamnetin**	MRM1	Y = (27.0601)X + (-0.0142331)	-1.42E-02	2.71E+01	0.99961	0.99981
	MRM2	Y = (6.61240)X + (-0.00204135)	-2.04E-03	6.61	0.99914	0.99957
**Kaempferol**	MRM1	Y = (5.67450)X + (0.000781581)	7.82E-04	5.67	0.99913	0.99956
	MRM2	Y = (4.59118)X + (0.00121183)	1.21E-03	4.59	0.99849	0.99925
**Quercetin dihydrate**	MRM1	Y = (40.9861)X + (-0.0311648)	-3.12E-02	4.10E+01	0.99879	0.99939
	MRM2	Y = (17.4478)X + (-0.0146218)	-1.46E-02	1.74E+01	0.99890	0.99945

y - dependent variable (response); m - slope of the line described by the equation, x - independent variable (concentration of the analyte), and b - y- coordinate of the y-intercept

Table S5. Intra- and inter-day precision at two concentration levels (100 and 1000 μg/L) for bilobalide, ginkgolides A, B, C, and J, isorhamnetin, kaempferol and quercetin dihydrate.

Compounds	Concentration (μg/L)	Intra-day	Inter-day
		RSD (%)	RSD (%)
Bilobalide	100	0.16	3.69
	1000	1.91	3.00
Ginkgolide A	100	1.76	3.26
	1000	2.09	4.16
Ginkgolide B	100	1.64	1.78
	1000	0.42	2.51
Ginkgolide C	100	0.32	3.57
	1000	2.69	3.73
Ginkgolide J	100	0.89	1.33
	1000	3.40	4.15
Isorhamnetin	100	3.46	5.50
	1000	1.35	5.19
Kaempferol	100	4.75	5.14
	1000	2.01	5.97
Quercetin dihydrate	100	0.60	7.35
	1000	1.26	8.75

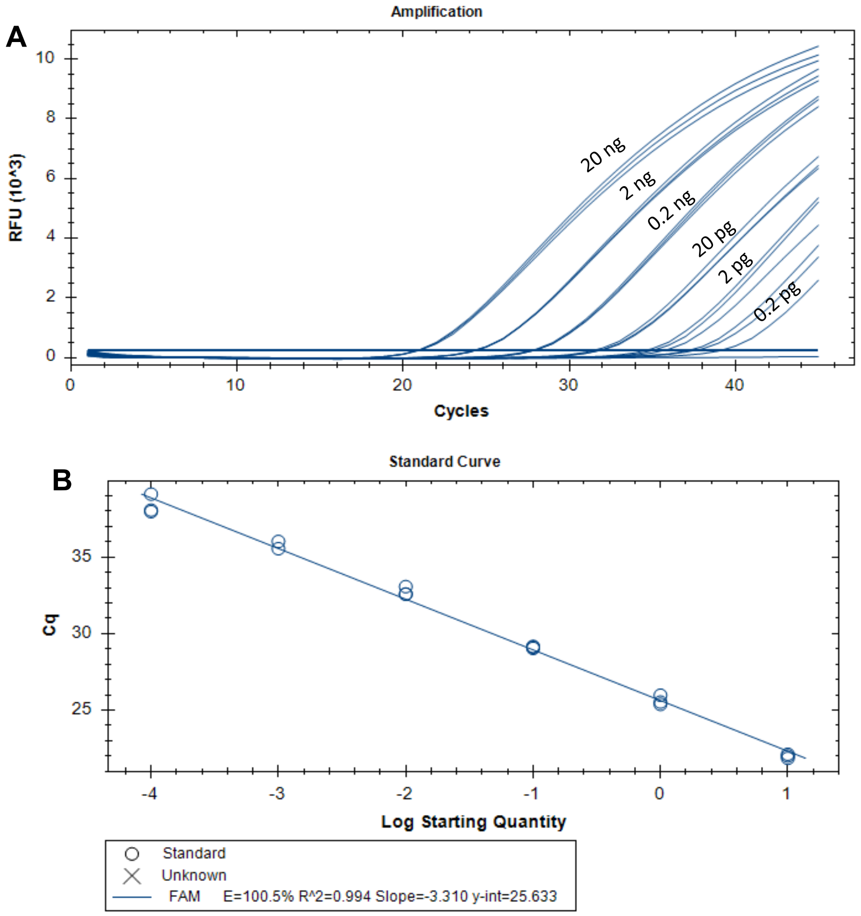

Figure S1. Amplification curves (A) and respective calibration curve (B) of a real-time PCR assay with a hydrolysis probe targeting ITS1 region of *G. biloba* using 10-fold serially diluted ginkgo DNA (20 ng to 0.2 pg) (*n*=3 replicates). Cq, cycle of quantification.

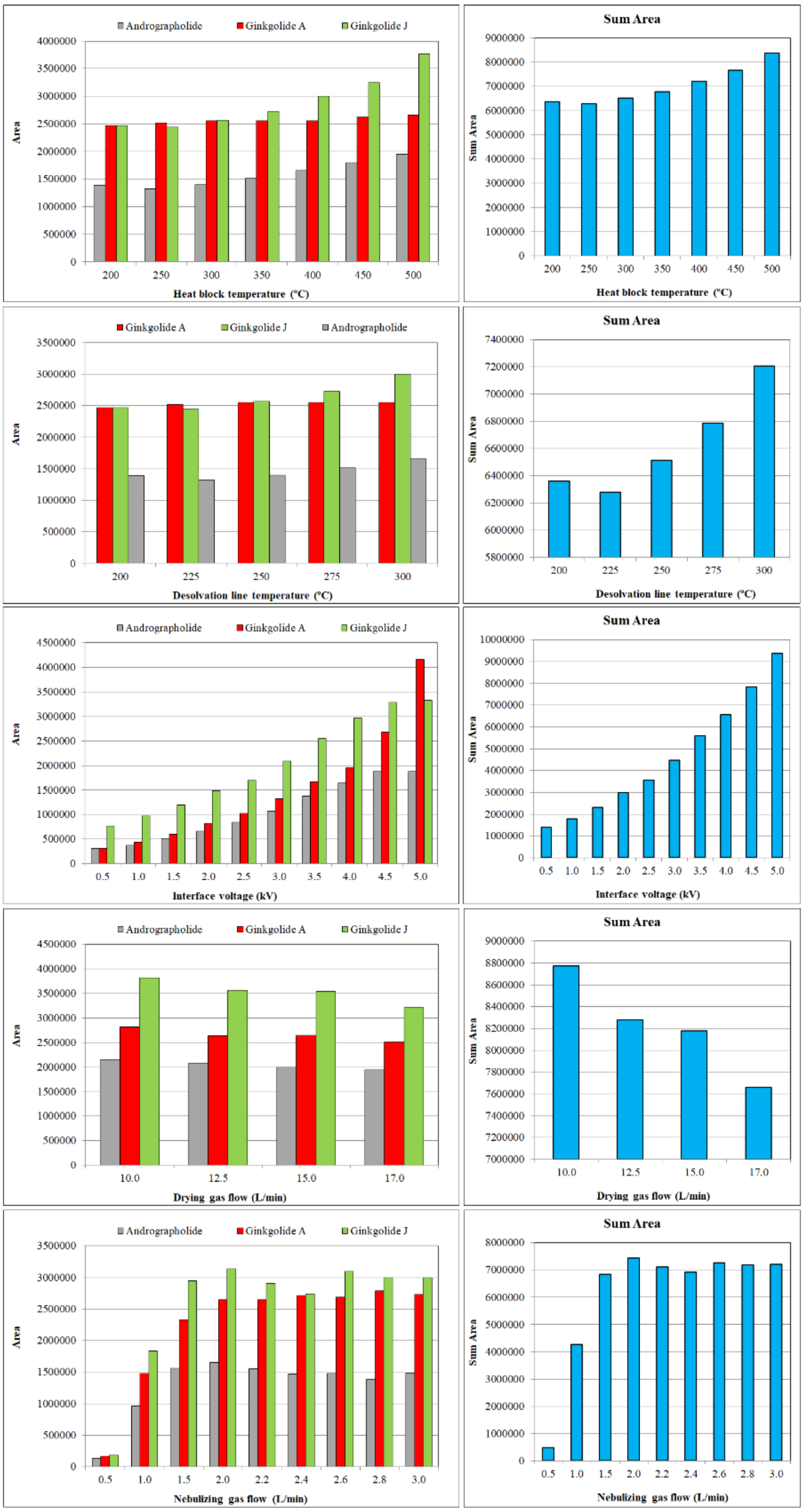

Figure S2. Areas obtained in the tests performed in the positive ionization mode for achieving the highest sensitivity, varying the heat block temperature, desolvation line temperature, interface voltage, drying gas flow, and nebulizing gas flow parameters for each compound and the sum area obtained for each ion source parameter.

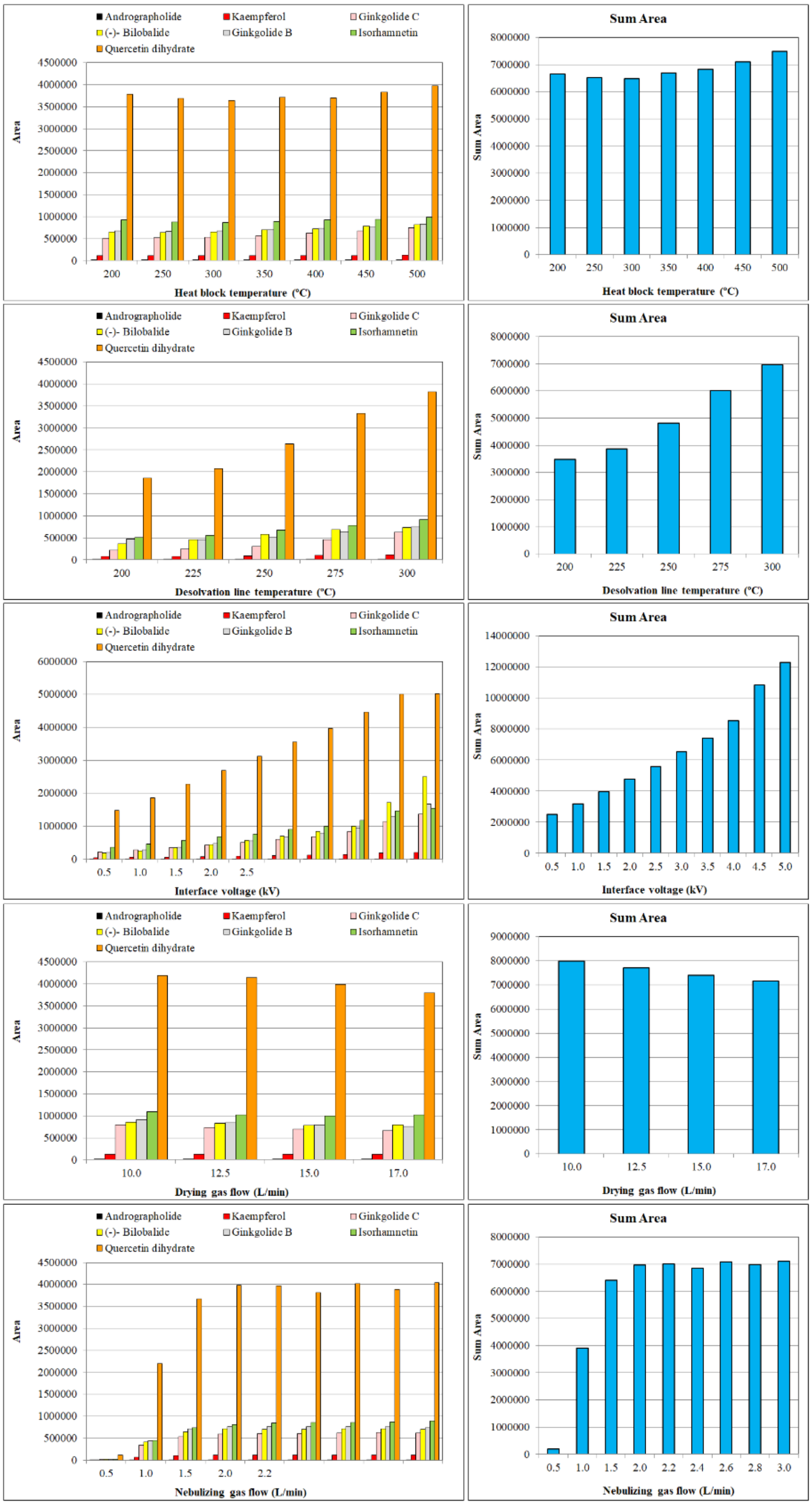

Figure S3. Areas obtained in the tests performed in the negative ionization mode for achieving the highest sensitivity, varying the heat block temperature, desolvation line temperature, interface voltage, drying gas flow, and nebulizing gas flow parameters for each compound and the sum area obtained for each ion source parameter.

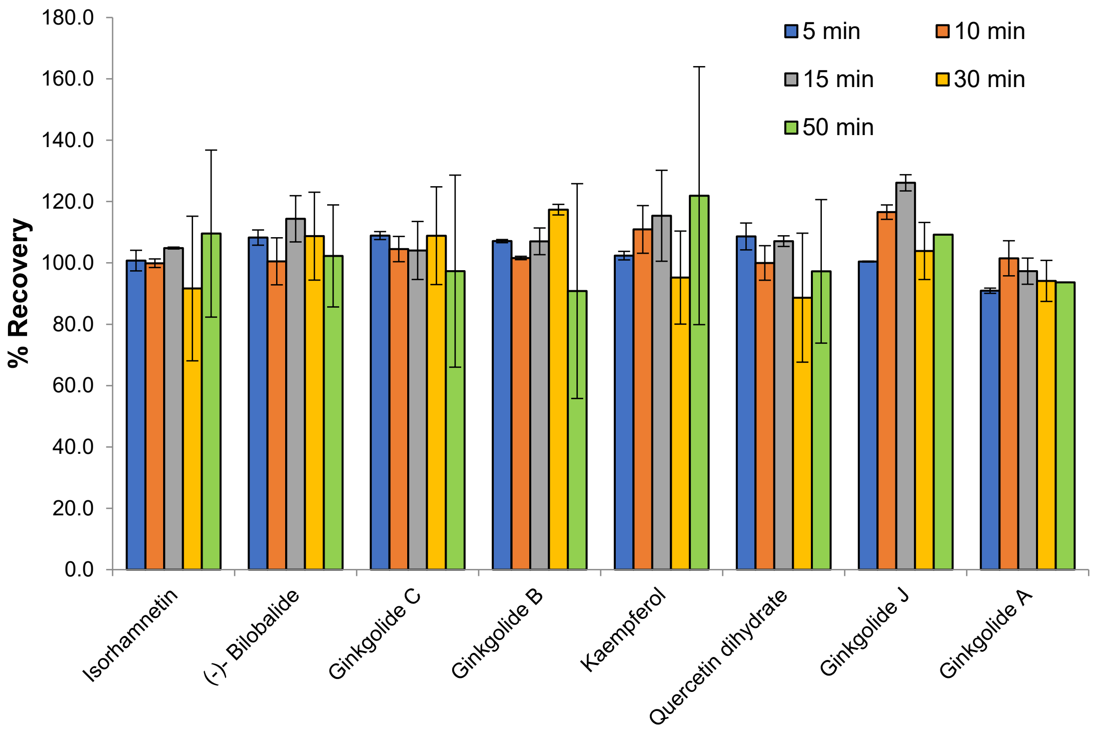

Figure S4. Recovery rates and RSD (*n*=2) obtained by testing different extraction times using an ultrasonic processor.

Solid samples	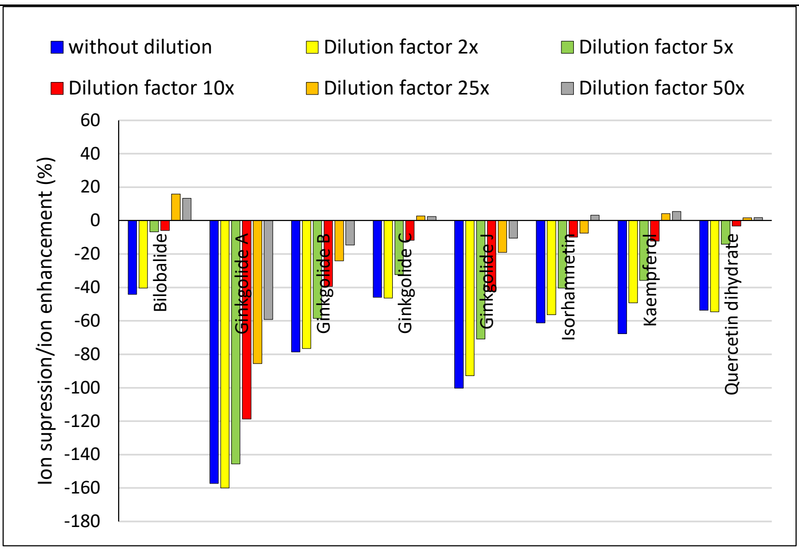
Liquid samples	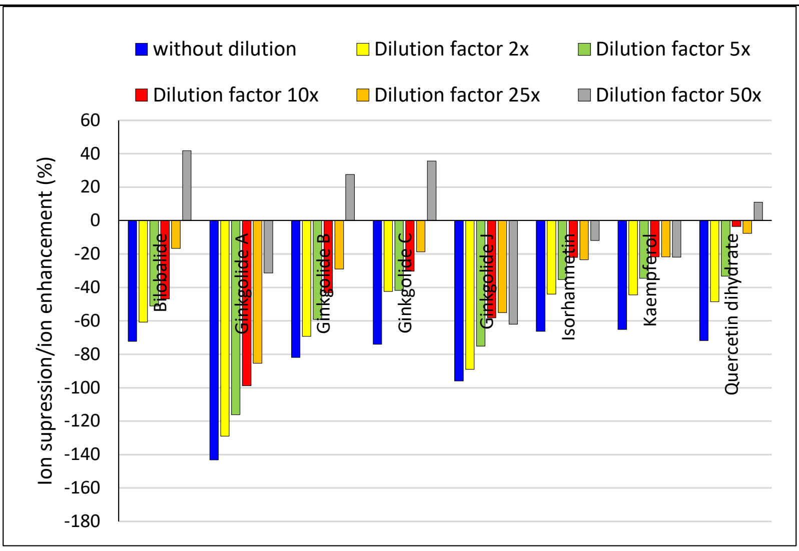
Lyophilised samples	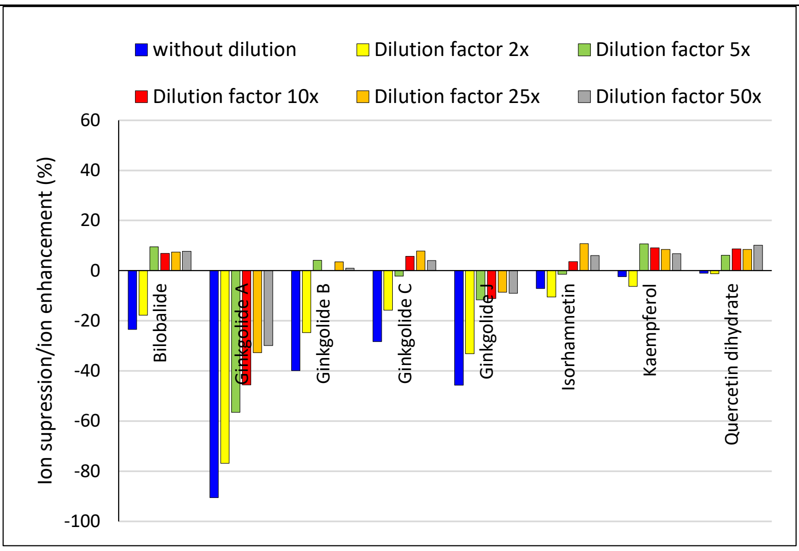

Figure S5. Matrix effect (ion suppression/ion enhancement) for solid and liquid PFS and lyophilized plant extracts without dilution and with various dilution factors (2x, 5x, 10x, 25x, 50x) for the studied compounds.
